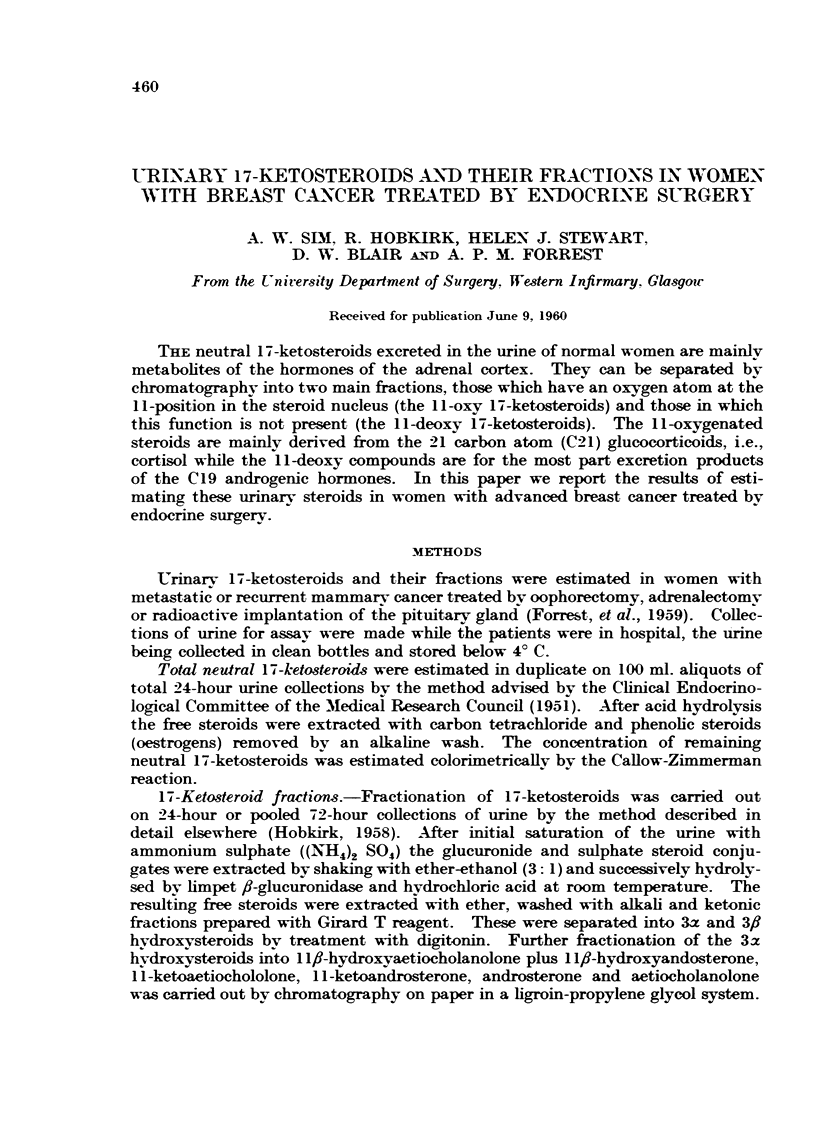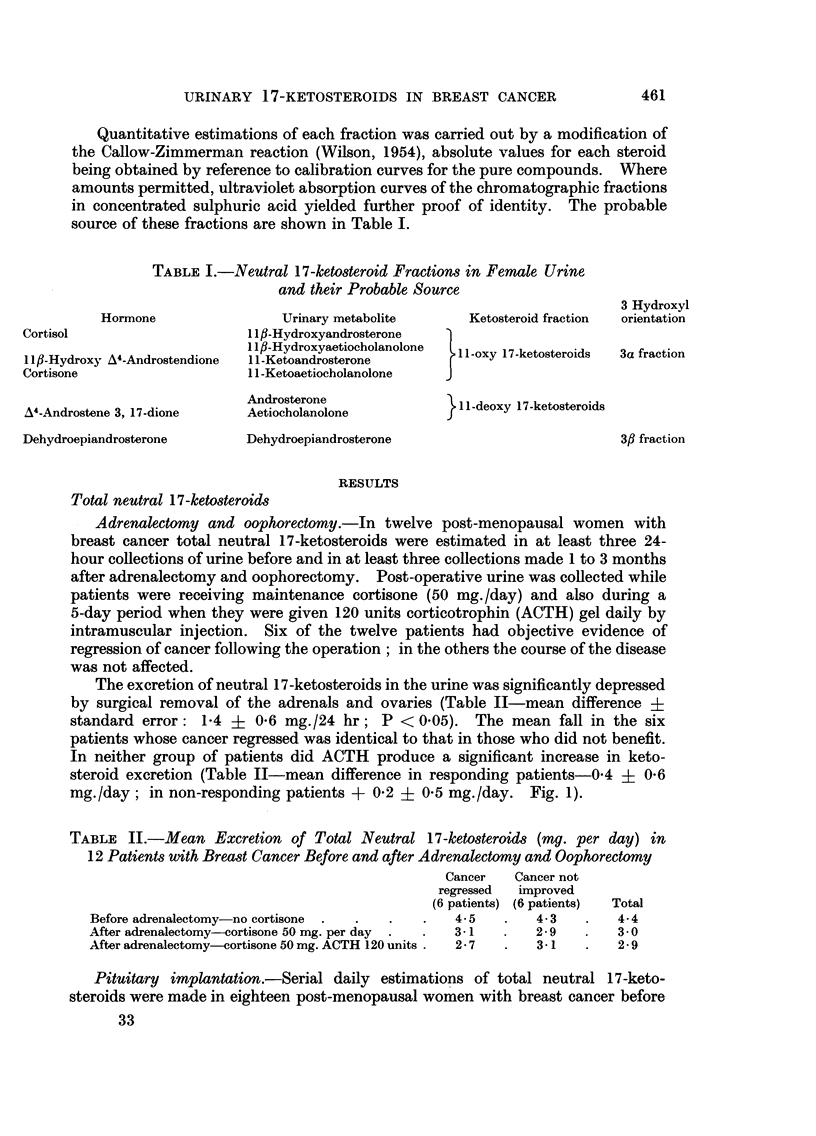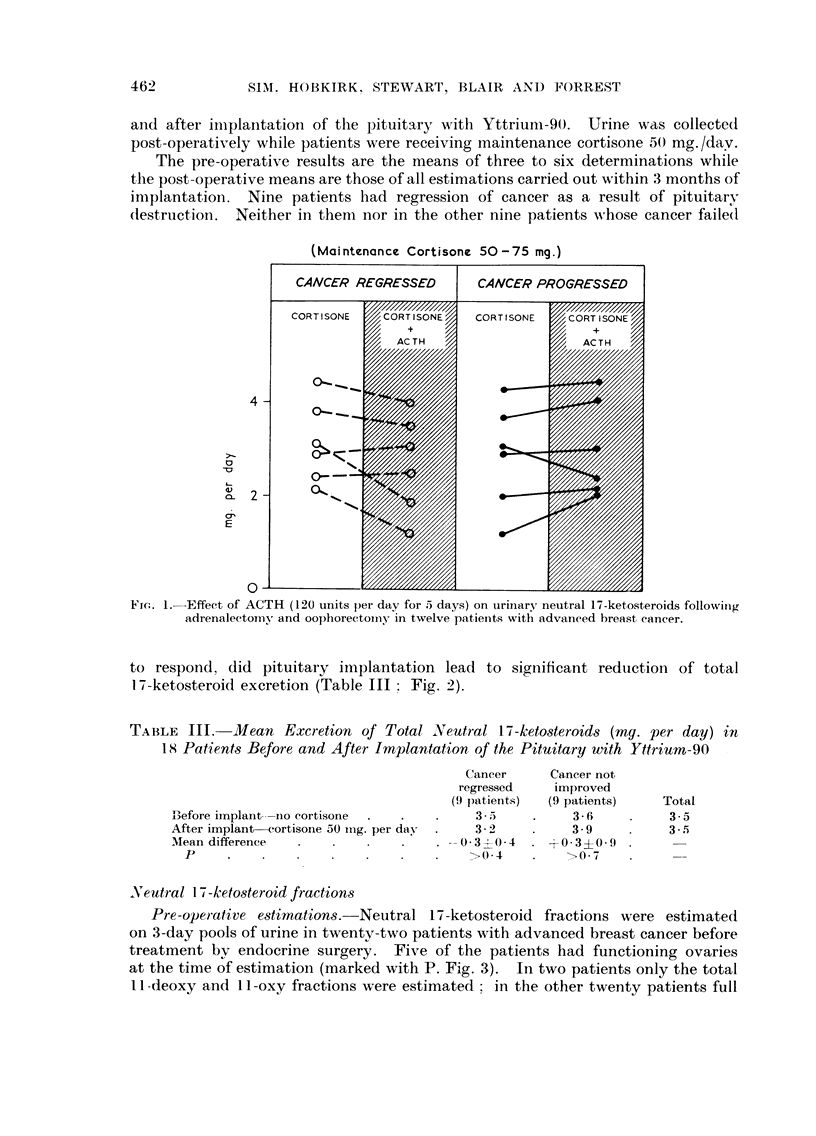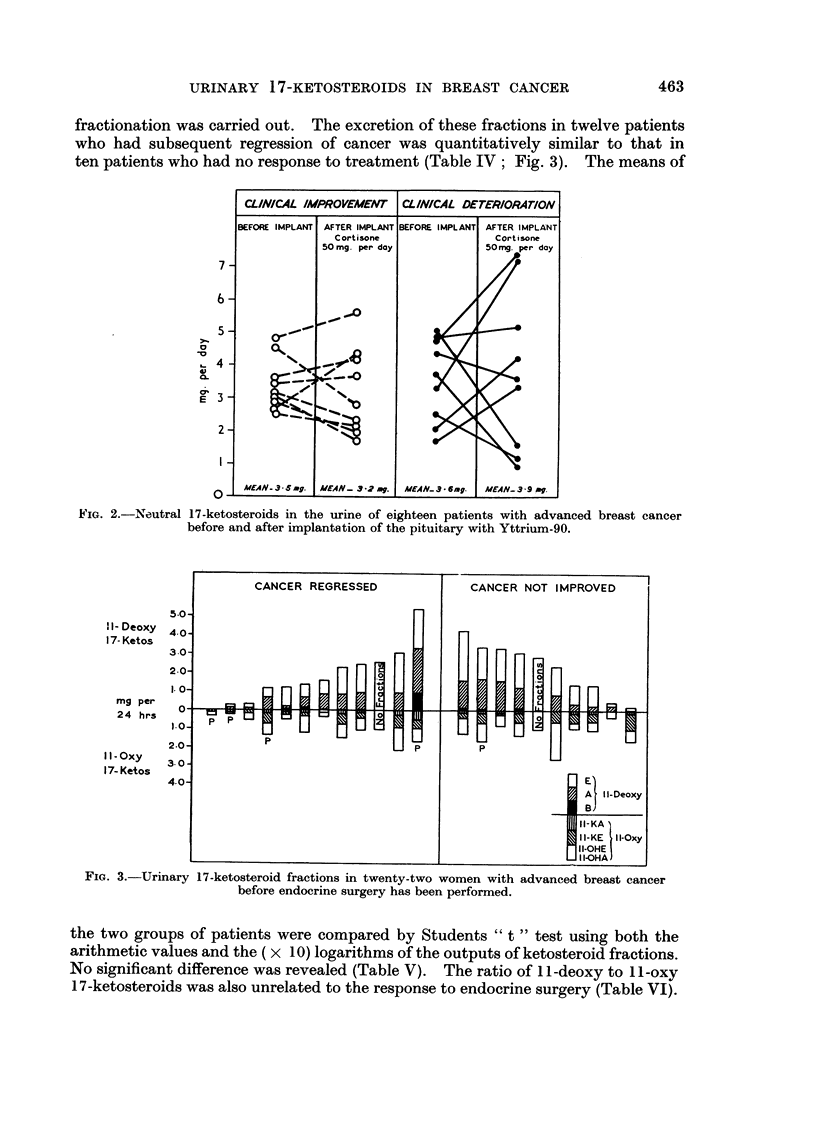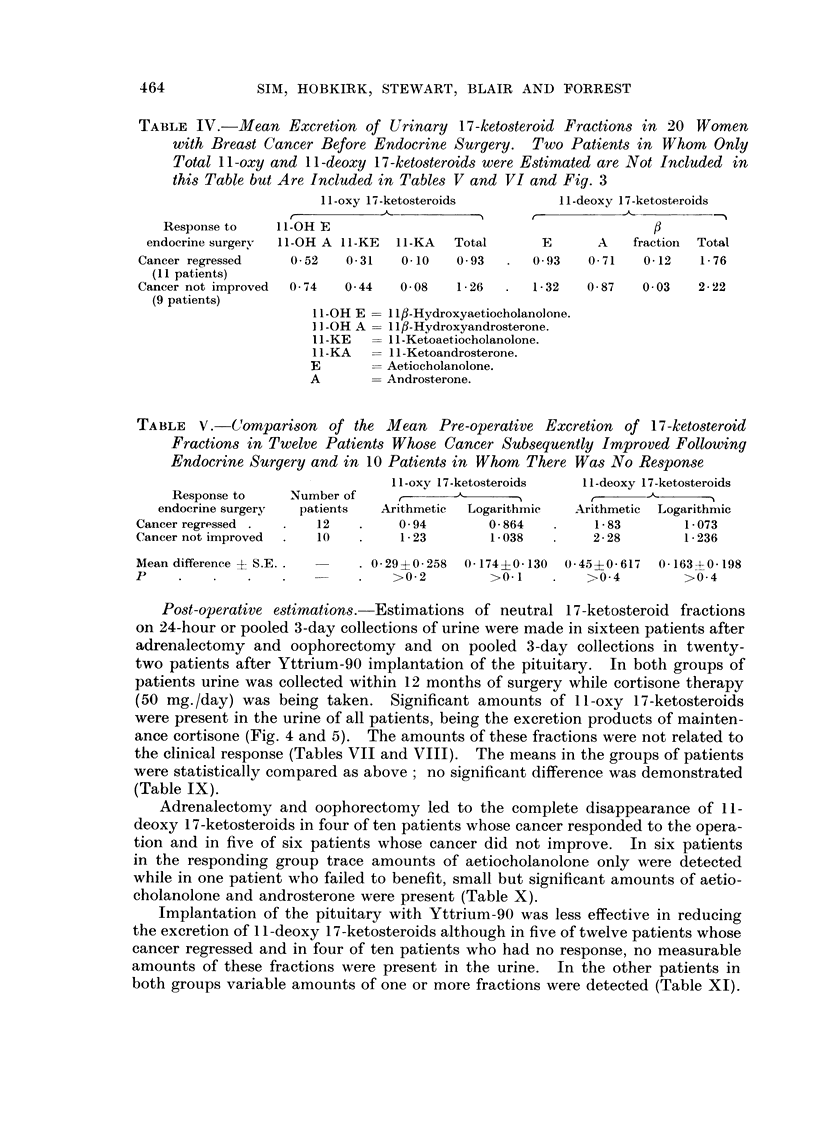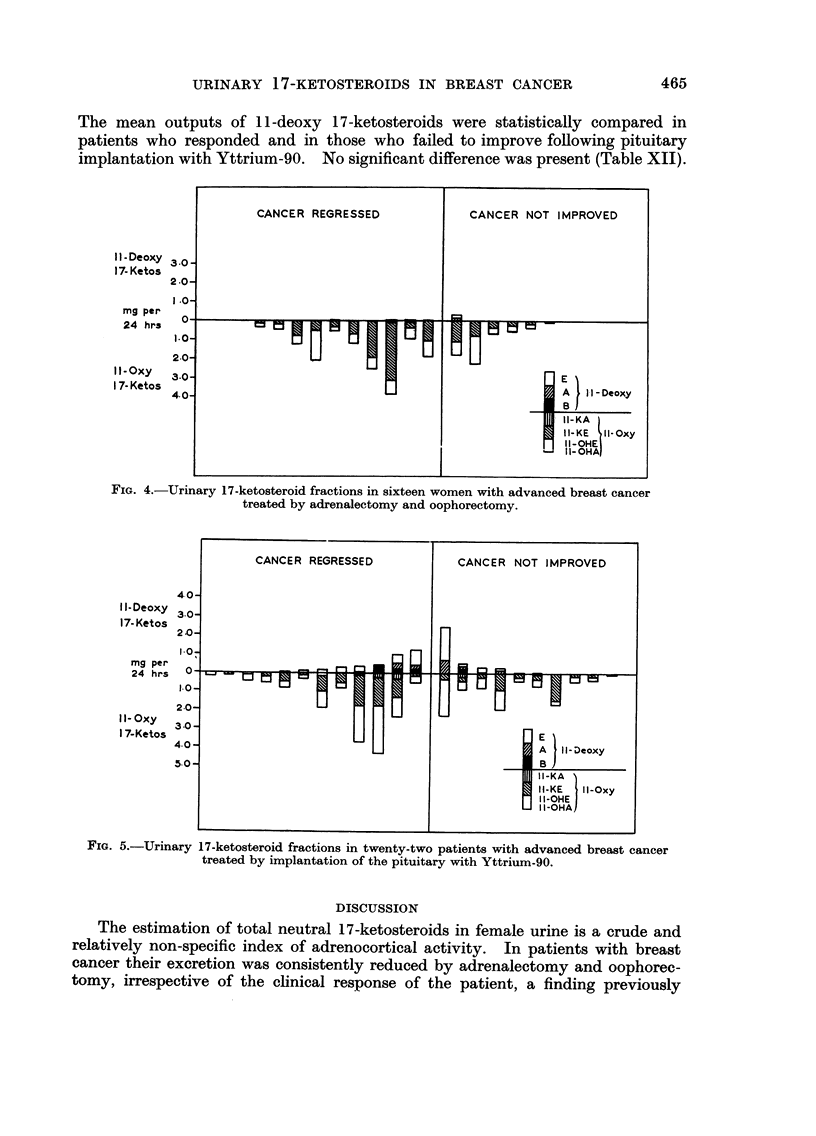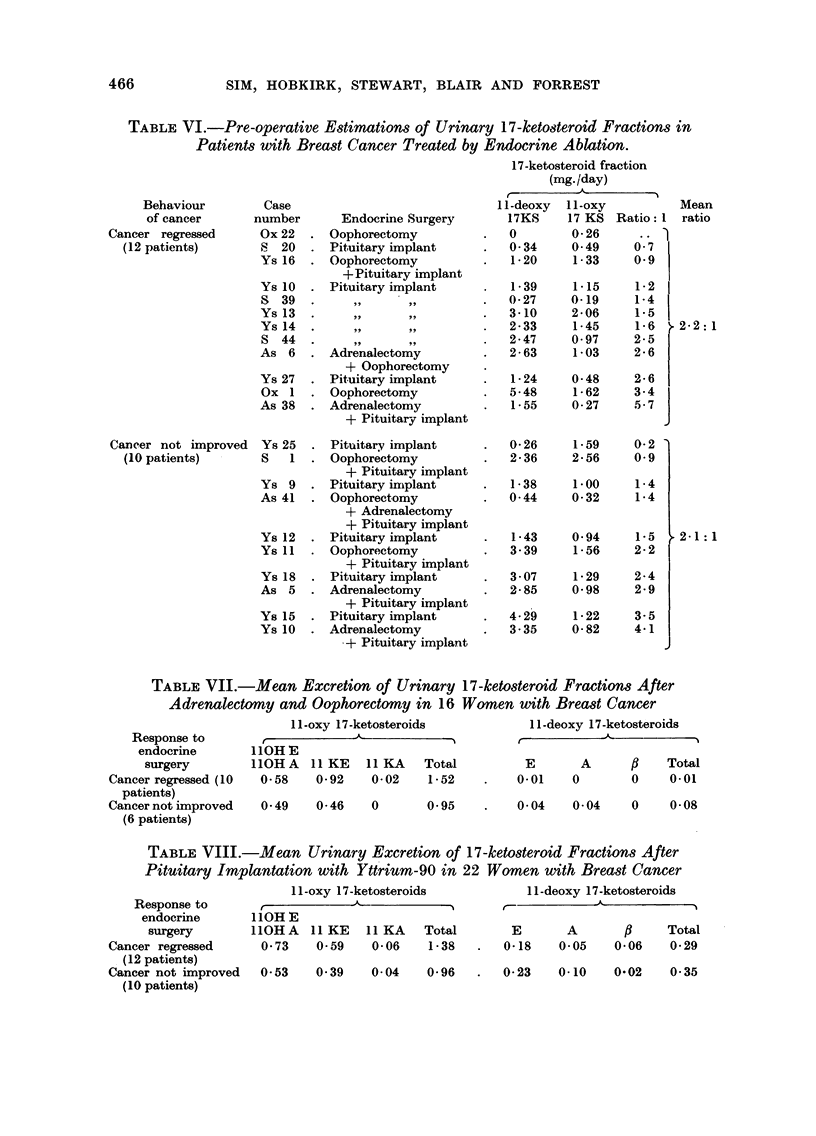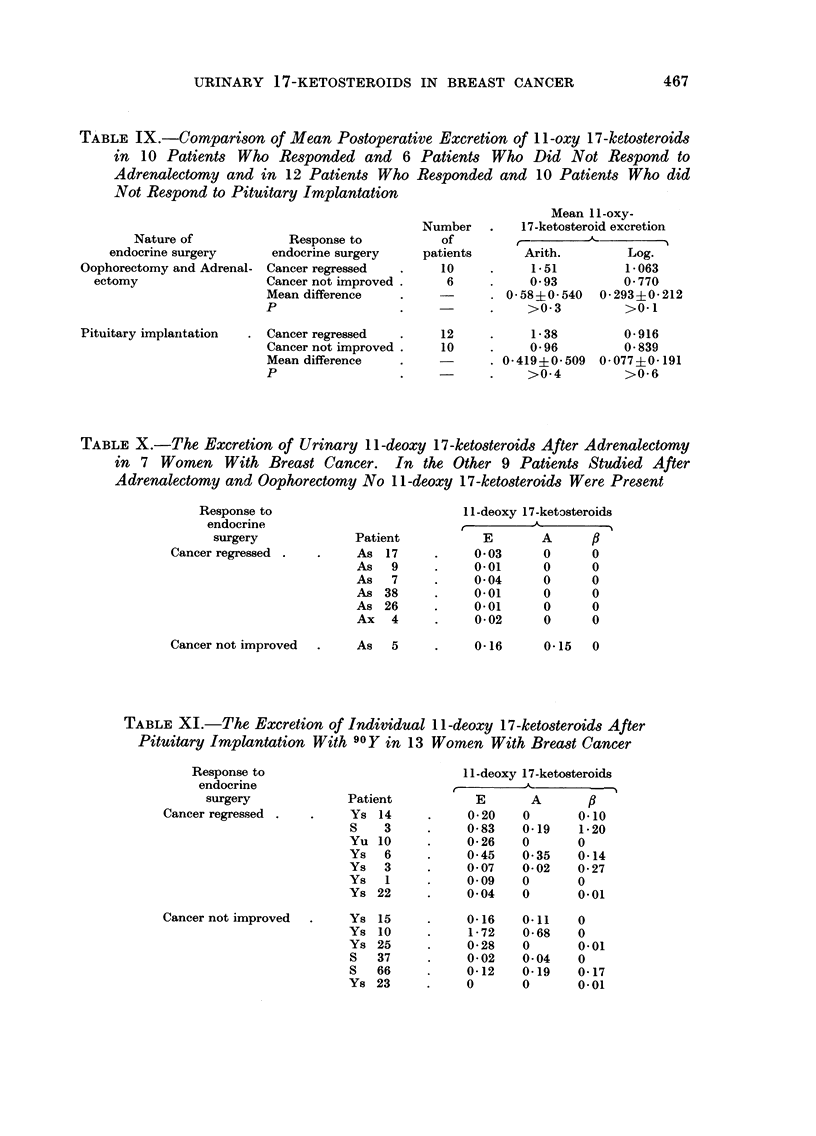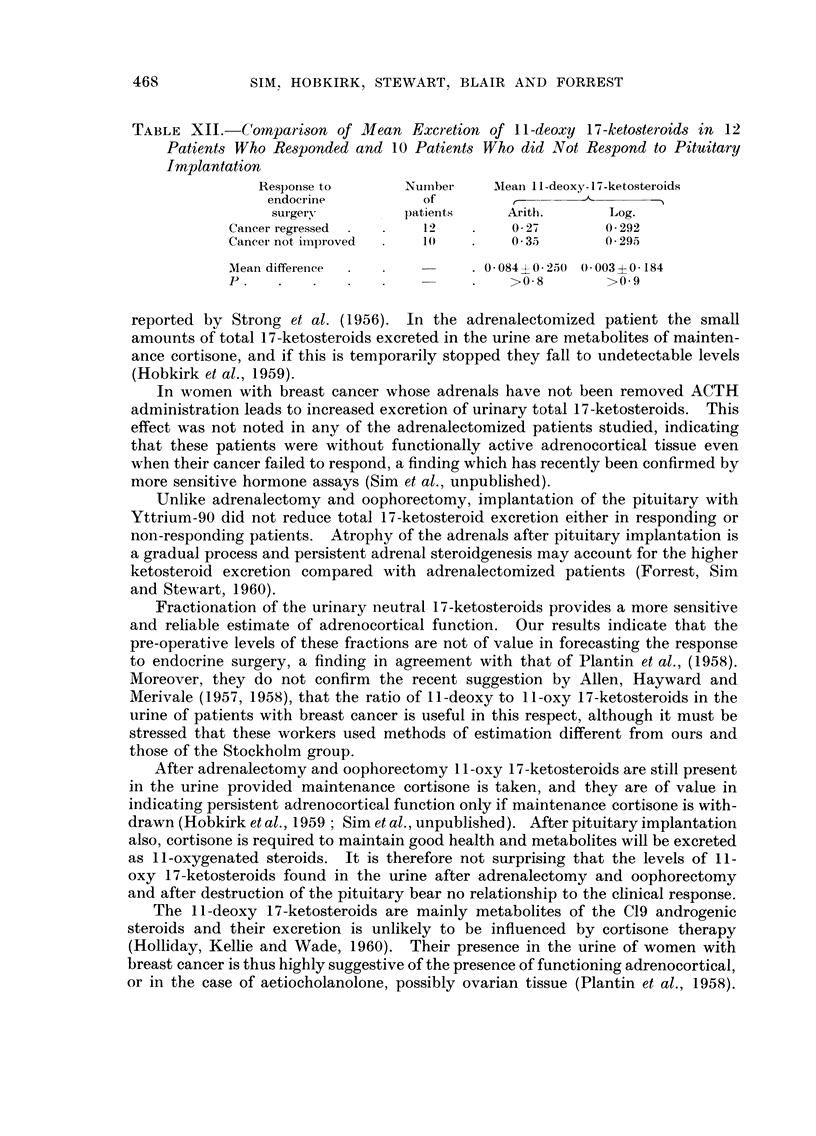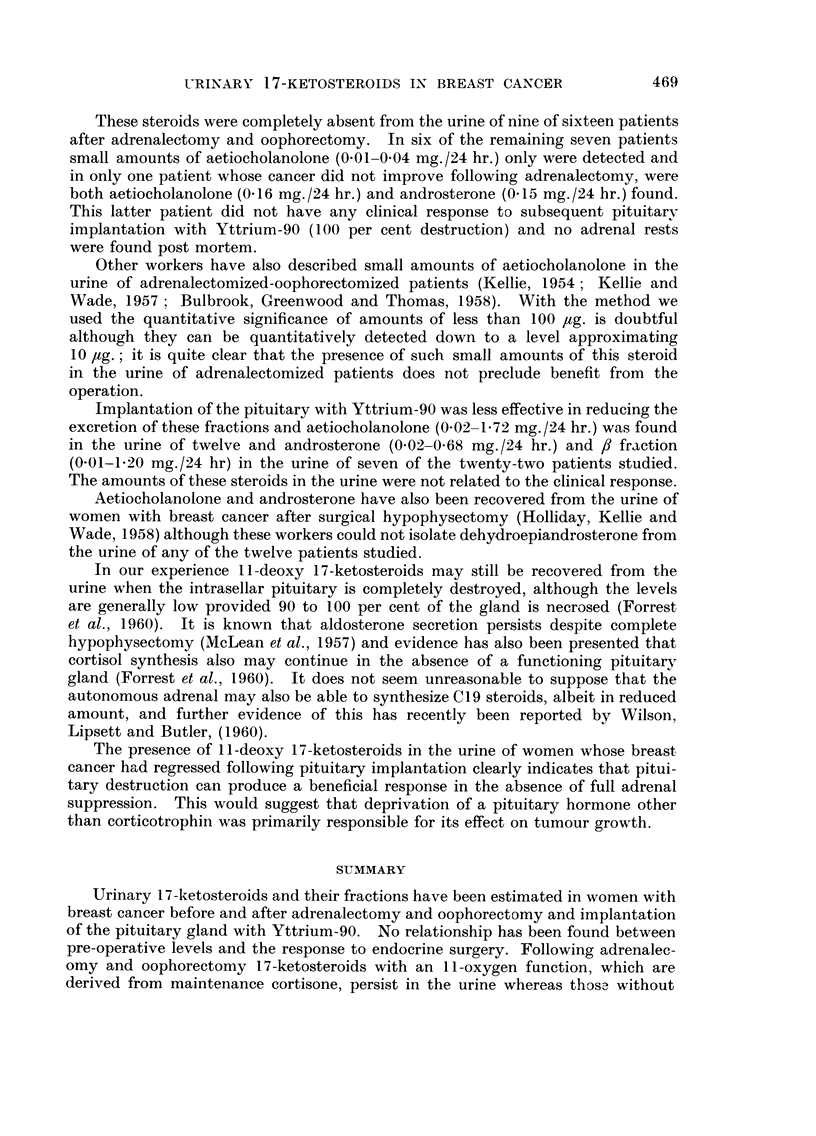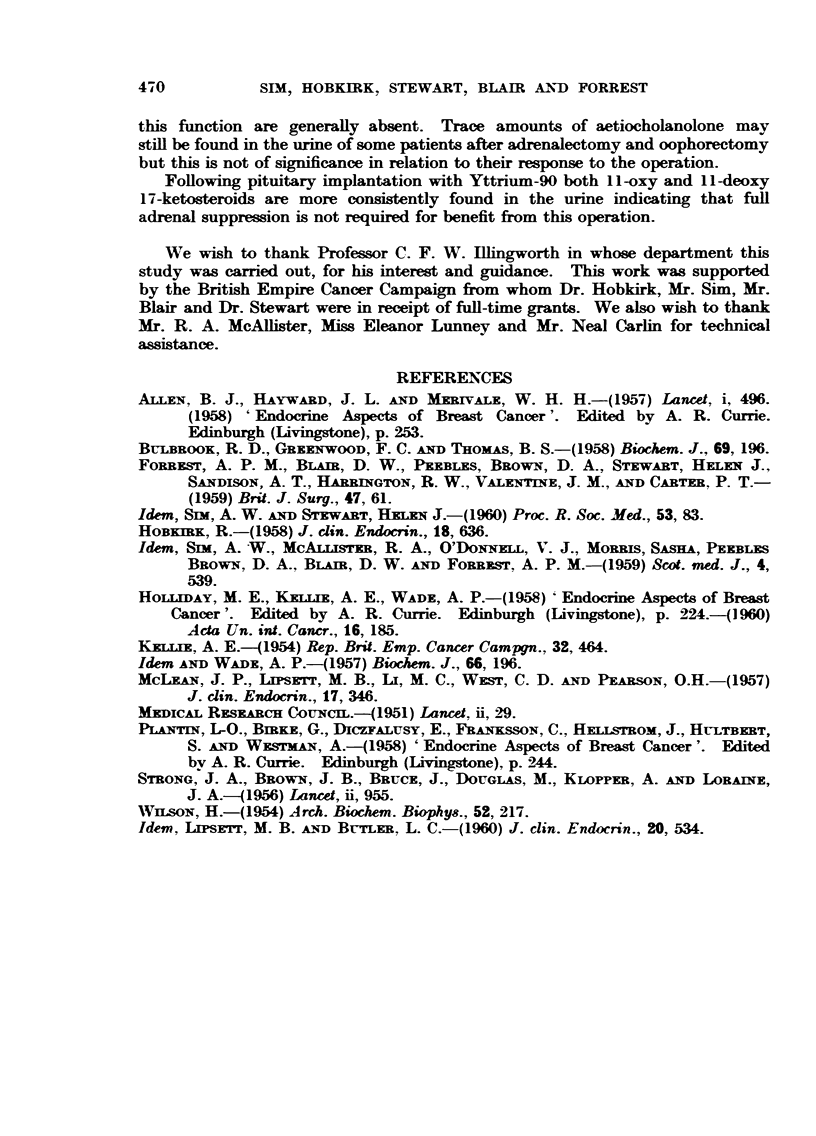# Urinary 17-Ketosteroids and their Fractions in Women with Breast Cancer Treated by Endocrine Surgery

**DOI:** 10.1038/bjc.1960.50

**Published:** 1960-09

**Authors:** A. W. Sim, R. Hobkirk, Helen J. Stewart, D. W. Blair, A. P. M. Forrest


### URINARY 17-KETOSTEROIDS AND THEIR FRACTIONS IN WOMEN WITH BREAST CANCER TREATED BY ENDOCRINE SURGERY

A. W. SIM, R. HOBKIRK, HELEN J. STEWART, D. W. BLAIR AND A. P. M. FORREST

*From the University Department of Surgery, Western Infirmary, Glasgow*

Received for publication June 9, 1960

THE neutral 17-ketosteroids excreted in the urine of normal women are mainly metabolites of the hormones of the adrenal cortex. They can be separated by chromatography into two main fractions, those which have an oxygen atom at the 11-position in the steroid nucleus (the 11-oxy 17-ketosteroids) and those in which this function is not present (the 11-deoxy 17-ketosteroids). The 11-oxygenated steroids are mainly derived from the 21 carbon atom (C21) glucocorticoids, i.e., cortisol while the 11-deoxy compounds are for the most part excretion products of the C19 androgenic hormones. In this paper we report the results of estimating these urinary steroids in women with advanced breast cancer treated by endocrine surgery.

### METHODS

Urinary 17-ketosteroids and their fractions were estimated in women with metastatic or recurrent mammary cancer treated by oophorectomy, adrenalectomy or radioactive implantation of the pituitary gland (Forrest, *et al.*, 1959). Collections of urine for assay were made while the patients were in hospital, the urine being collected in clean bottles and stored below 4° C.

*Total neutral 17-ketosteroids* were estimated in duplicate on 100 ml. aliquots of total 24-hour urine collections by the method advised by the Clinical Endocrinological Committee of the Medical Research Council (1951). After acid hydrolysis the free steroids were extracted with carbon tetrachloride and phenolic steroids (oestrogens) removed by an alkaline wash. The concentration of remaining neutral 17-ketosteroids was estimated colorimetrically by the Callow-Zimmerman reaction.

*17-Ketosteroid fractions.*—Fractionation of 17-ketosteroids was carried out on 24-hour or pooled 72-hour collections of urine by the method described in detail elsewhere (Hobkirk, 1958). After initial saturation of the urine with ammonium sulphate ($(NH_4)_2\ SO_4$) the glucuronide and sulphate steroid conjugates were extracted by shaking with ether-ethanol (3 : 1) and successively hydrolysed by limpet $\beta$-glucuronidase and hydrochloric acid at room temperature. The resulting free steroids were extracted with ether, washed with alkali and ketonic fractions prepared with Girard T reagent. These were separated into $3\alpha$ and $3\beta$ hydroxysteroids by treatment with digitonin. Further fractionation of the $3\alpha$ hydroxysteroids into $11\beta$-hydroxyaetiocholanolone plus $11\beta$-hydroxyandosterone, 11-ketoaetiochololone, 11-ketoandrosterone, androsterone and aetiocholanolone was carried out by chromatography on paper in a ligroin-propylene glycol system.

Quantitative estimations of each fraction was carried out by a modification of the Callow-Zimmerman reaction (Wilson, 1954), absolute values for each steroid being obtained by reference to calibration curves for the pure compounds. Where amounts permitted, ultraviolet absorption curves of the chromatographic fractions in concentrated sulphuric acid yielded further proof of identity. The probable source of these fractions are shown in Table I.

TABLE I.—*Neutral 17-ketosteroid Fractions in Female Urine and their Probable Source*

| Hormone | Urinary metabolite | Ketosteroid fraction | 3 Hydroxyl orientation |
|---|---|---|---|
| Cortisol | 11β-Hydroxyandrosterone | 11-oxy 17-ketosteroids | 3α fraction |
| | 11β-Hydroxyaetiocholanolone | | |
| 11β-Hydroxy $\Delta^4$-Androstendione | 11-Ketoandrosterone | | |
| Cortisone | 11-Ketoaetiocholanolone | | |
| | Androsterone | 11-deoxy 17-ketosteroids | |
| $\Delta^4$-Androstene 3, 17-dione | Aetiocholanolone | | |
| Dehydroepiandrosterone | Dehydroepiandrosterone | | 3β fraction |

RESULTS

*Total neutral 17-ketosteroids*

*Adrenalectomy and oophorectomy.*—In twelve post-menopausal women with breast cancer total neutral 17-ketosteroids were estimated in at least three 24-hour collections of urine before and in at least three collections made 1 to 3 months after adrenalectomy and oophorectomy. Post-operative urine was collected while patients were receiving maintenance cortisone (50 mg./day) and also during a 5-day period when they were given 120 units corticotrophin (ACTH) gel daily by intramuscular injection. Six of the twelve patients had objective evidence of regression of cancer following the operation ; in the others the course of the disease was not affected.

The excretion of neutral 17-ketosteroids in the urine was significantly depressed by surgical removal of the adrenals and ovaries (Table II—mean difference $\pm$ standard error : $1\cdot4 \pm 0\cdot6$ mg./24 hr ; $P < 0\cdot05$). The mean fall in the six patients whose cancer regressed was identical to that in those who did not benefit. In neither group of patients did ACTH produce a significant increase in keto-steroid excretion (Table II—mean difference in responding patients—$0\cdot4 \pm 0\cdot6$ mg./day ; in non-responding patients $+ 0\cdot2 \pm 0\cdot5$ mg./day. Fig. 1).

TABLE II.—*Mean Excretion of Total Neutral 17-ketosteroids (mg. per day) in 12 Patients with Breast Cancer Before and after Adrenalectomy and Oophorectomy*

| | Cancer regressed (6 patients) | Cancer not improved (6 patients) | Total |
|---|---|---|---|
| Before adrenalectomy—no cortisone | 4·5 | 4·3 | 4·4 |
| After adrenalectomy—cortisone 50 mg. per day | 3·1 | 2·9 | 3·0 |
| After adrenalectomy—cortisone 50 mg. ACTH 120 units | 2·7 | 3·1 | 2·9 |

*Pituitary implantation.*—Serial daily estimations of total neutral 17-keto-steroids were made in eighteen post-menopausal women with breast cancer before and after implantation of the pituitary with Yttrium-90. Urine was collected post-operatively while patients were receiving maintenance cortisone 50 mg./day.

The pre-operative results are the means of three to six determinations while the post-operative means are those of all estimations carried out within 3 months of implantation. Nine patients had regression of cancer as a result of pituitary destruction. Neither in them nor in the other nine patients whose cancer failed

FIG. 1.—Effect of ACTH (120 units per day for 5 days) on urinary neutral 17-ketosteroids following adrenalectomy and oophorectomy in twelve patients with advanced breast cancer.

to respond, did pituitary implantation lead to significant reduction of total 17-ketosteroid excretion (Table III ; Fig. 2).

TABLE III.—*Mean Excretion of Total Neutral 17-ketosteroids (mg. per day) in 18 Patients Before and After Implantation of the Pituitary with Yttrium-90*

| | Cancer regressed (9 patients) | Cancer not improved (9 patients) | Total |
|---|---|---|---|
| Before implant—no cortisone | 3·5 | 3·6 | 3·5 |
| After implant—cortisone 50 mg. per day | 3·2 | 3·9 | 3·5 |
| Mean difference | −0·3±0·4 | +0·3±0·9 | — |
| *P* | >0·4 | >0·7 | — |

*Neutral 17-ketosteroid fractions*

*Pre-operative estimations.*—Neutral 17-ketosteroid fractions were estimated on 3-day pools of urine in twenty-two patients with advanced breast cancer before treatment by endocrine surgery. Five of the patients had functioning ovaries at the time of estimation (marked with P. Fig. 3). In two patients only the total 11-deoxy and 11-oxy fractions were estimated ; in the other twenty patients full fractionation was carried out. The excretion of these fractions in twelve patients who had subsequent regression of cancer was quantitatively similar to that in ten patients who had no response to treatment (Table IV ; Fig. 3). The means of

FIG. 2.—Neutral 17-ketosteroids in the urine of eighteen patients with advanced breast cancer before and after implantation of the pituitary with Yttrium-90.

FIG. 3.—Urinary 17-ketosteroid fractions in twenty-two women with advanced breast cancer before endocrine surgery has been performed.

the two groups of patients were compared by Students " t " test using both the arithmetic values and the ( × 10) logarithms of the outputs of ketosteroid fractions. No significant difference was revealed (Table V). The ratio of 11-deoxy to 11-oxy 17-ketosteroids was also unrelated to the response to endocrine surgery (Table VI).

TABLE IV.—*Mean Excretion of Urinary* 17-*ketosteroid Fractions in* 20 *Women with Breast Cancer Before Endocrine Surgery. Two Patients in Whom Only Total* 11-*oxy and* 11-*deoxy* 17-*ketosteroids were Estimated are Not Included in this Table but Are Included in Tables V and VI and Fig.* 3

| Response to endocrine surgery | 11-oxy 17-ketosteroids: 11-OH E 11-OH A | 11-KE | 11-KA | Total | | 11-deoxy 17-ketosteroids: E | A | β fraction | Total |
|---|---|---|---|---|---|---|---|---|---|
| Cancer regressed (11 patients) | 0·52 | 0·31 | 0·10 | 0·93 | . | 0·93 | 0·71 | 0·12 | 1·76 |
| Cancer not improved (9 patients) | 0·74 | 0·44 | 0·08 | 1·26 | . | 1·32 | 0·87 | 0·03 | 2·22 |

11-OH E = 11β-Hydroxyaetiocholanolone.

11-OH A = 11β-Hydroxyandrosterone.

11-KE = 11-Ketoaetiocholanolone.

11-KA = 11-Ketoandrosterone.

E = Aetiocholanolone.

A = Androsterone.

TABLE V.—*Comparison of the Mean Pre-operative Excretion of* 17-*ketosteroid Fractions in Twelve Patients Whose Cancer Subsequently Improved Following Endocrine Surgery and in* 10 *Patients in Whom There Was No Response*

| Response to endocrine surgery | Number of patients | | 11-oxy 17-ketosteroids: Arithmetic | Logarithmic | | 11-deoxy 17-ketosteroids: Arithmetic | Logarithmic |
|---|---|---|---|---|---|---|---|
| Cancer regressed . . | 12 | . | 0·94 | 0·864 | . | 1·83 | 1·073 |
| Cancer not improved . | 10 | . | 1·23 | 1·038 | . | 2·28 | 1·236 |
| Mean difference ± S.E. . | — | . | 0·29±0·258 | 0·174±0·130 | | 0·45±0·617 | 0·163±0·198 |
| *P* . . . . . | — | . | >0·2 | >0·1 | . | >0·4 | >0·4 |

*Post-operative estimations.*—Estimations of neutral 17-ketosteroid fractions on 24-hour or pooled 3-day collections of urine were made in sixteen patients after adrenalectomy and oophorectomy and on pooled 3-day collections in twenty-two patients after Yttrium-90 implantation of the pituitary. In both groups of patients urine was collected within 12 months of surgery while cortisone therapy (50 mg./day) was being taken. Significant amounts of 11-oxy 17-ketosteroids were present in the urine of all patients, being the excretion products of maintenance cortisone (Fig. 4 and 5). The amounts of these fractions were not related to the clinical response (Tables VII and VIII). The means in the groups of patients were statistically compared as above ; no significant difference was demonstrated (Table IX).

Adrenalectomy and oophorectomy led to the complete disappearance of 11-deoxy 17-ketosteroids in four of ten patients whose cancer responded to the operation and in five of six patients whose cancer did not improve. In six patients in the responding group trace amounts of aetiocholanolone only were detected while in one patient who failed to benefit, small but significant amounts of aetiocholanolone and androsterone were present (Table X).

Implantation of the pituitary with Yttrium-90 was less effective in reducing the excretion of 11-deoxy 17-ketosteroids although in five of twelve patients whose cancer regressed and in four of ten patients who had no response, no measurable amounts of these fractions were present in the urine. In the other patients in both groups variable amounts of one or more fractions were detected (Table XI).

The mean outputs of 11-deoxy 17-ketosteroids were statistically compared in patients who responded and in those who failed to improve following pituitary implantation with Yttrium-90. No significant difference was present (Table XII).

FIG. 4.—Urinary 17-ketosteroid fractions in sixteen women with advanced breast cancer treated by adrenalectomy and oophorectomy.

FIG. 5.—Urinary 17-ketosteroid fractions in twenty-two patients with advanced breast cancer treated by implantation of the pituitary with Yttrium-90.

DISCUSSION

The estimation of total neutral 17-ketosteroids in female urine is a crude and relatively non-specific index of adrenocortical activity. In patients with breast cancer their excretion was consistently reduced by adrenalectomy and oophorectomy, irrespective of the clinical response of the patient, a finding previously

TABLE VI.—*Pre-operative Estimations of Urinary 17-ketosteroid Fractions in Patients with Breast Cancer Treated by Endocrine Ablation.*

| Behaviour of cancer | Case number | Endocrine Surgery | 17-ketosteroid fraction (mg./day) 11-deoxy 17KS | 11-oxy 17 KS | Ratio : 1 | Mean ratio |
|---|---|---|---|---|---|---|
| Cancer regressed (12 patients) | Ox 22 | Oophorectomy | 0 | 0·26 | .. | 2·2 : 1 |
| | S 20 | Pituitary implant | 0·34 | 0·49 | 0·7 | |
| | Ys 16 | Oophorectomy + Pituitary implant | 1·20 | 1·33 | 0·9 | |
| | Ys 10 | Pituitary implant | 1·39 | 1·15 | 1·2 | |
| | S 39 | ,, ,, | 0·27 | 0·19 | 1·4 | |
| | Ys 13 | ,, ,, | 3·10 | 2·06 | 1·5 | |
| | Ys 14 | ,, ,, | 2·33 | 1·45 | 1·6 | |
| | S 44 | ,, ,, | 2·47 | 0·97 | 2·5 | |
| | As 6 | Adrenalectomy + Oophorectomy | 2·63 | 1·03 | 2·6 | |
| | Ys 27 | Pituitary implant | 1·24 | 0·48 | 2·6 | |
| | Ox 1 | Oophorectomy | 5·48 | 1·62 | 3·4 | |
| | As 38 | Adrenalectomy + Pituitary implant | 1·55 | 0·27 | 5·7 | |
| Cancer not improved (10 patients) | Ys 25 | Pituitary implant | 0·26 | 1·59 | 0·2 | 2·1 : 1 |
| | S 1 | Oophorectomy + Pituitary implant | 2·36 | 2·56 | 0·9 | |
| | Ys 9 | Pituitary implant | 1·38 | 1·00 | 1·4 | |
| | As 41 | Oophorectomy + Adrenalectomy + Pituitary implant | 0·44 | 0·32 | 1·4 | |
| | Ys 12 | Pituitary implant | 1·43 | 0·94 | 1·5 | |
| | Ys 11 | Oophorectomy + Pituitary implant | 3·39 | 1·56 | 2·2 | |
| | Ys 18 | Pituitary implant | 3·07 | 1·29 | 2·4 | |
| | As 5 | Adrenalectomy + Pituitary implant | 2·85 | 0·98 | 2·9 | |
| | Ys 15 | Pituitary implant | 4·29 | 1·22 | 3·5 | |
| | Ys 10 | Adrenalectomy + Pituitary implant | 3·35 | 0·82 | 4·1 | |

TABLE VII.—*Mean Excretion of Urinary 17-ketosteroid Fractions After Adrenalectomy and Oophorectomy in 16 Women with Breast Cancer*

| Response to endocrine surgery | 11-oxy 17-ketosteroids 11OH E 11OH A | 11 KE | 11 KA | Total | 11-deoxy 17-ketosteroids E | A | β | Total |
|---|---|---|---|---|---|---|---|---|
| Cancer regressed (10 patients) | 0·58 | 0·92 | 0·02 | 1·52 | 0·01 | 0 | 0 | 0·01 |
| Cancer not improved (6 patients) | 0·49 | 0·46 | 0 | 0·95 | 0·04 | 0·04 | 0 | 0·08 |

TABLE VIII.—*Mean Urinary Excretion of 17-ketosteroid Fractions After Pituitary Implantation with Yttrium-90 in 22 Women with Breast Cancer*

| Response to endocrine surgery | 11-oxy 17-ketosteroids 11OH E 11OH A | 11 KE | 11 KA | Total | 11-deoxy 17-ketosteroids E | A | β | Total |
|---|---|---|---|---|---|---|---|---|
| Cancer regressed (12 patients) | 0·73 | 0·59 | 0·06 | 1·38 | 0·18 | 0·05 | 0·06 | 0·29 |
| Cancer not improved (10 patients) | 0·53 | 0·39 | 0·04 | 0·96 | 0·23 | 0·10 | 0·02 | 0·35 |

TABLE IX.—*Comparison of Mean Postoperative Excretion of* 11-*oxy* 17-*ketosteroids in* 10 *Patients Who Responded and* 6 *Patients Who Did Not Respond to Adrenalectomy and in* 12 *Patients Who Responded and* 10 *Patients Who did Not Respond to Pituitary Implantation*

| Nature of endocrine surgery | Response to endocrine surgery | Number of patients | Mean 11-oxy-17-ketosteroid excretion: Arith. | Log. |
|---|---|---|---|---|
| Oophorectomy and Adrenalectomy | Cancer regressed | 10 | 1·51 | 1·063 |
| | Cancer not improved | 6 | 0·93 | 0·770 |
| | Mean difference | — | 0·58±0·540 | 0·293±0·212 |
| | *P* | — | >0·3 | >0·1 |
| Pituitary implantation | Cancer regressed | 12 | 1·38 | 0·916 |
| | Cancer not improved | 10 | 0·96 | 0·839 |
| | Mean difference | — | 0·419±0·509 | 0·077±0·191 |
| | *P* | — | >0·4 | >0·6 |

TABLE X.—*The Excretion of Urinary* 11-*deoxy* 17-*ketosteroids After Adrenalectomy in* 7 *Women With Breast Cancer. In the Other* 9 *Patients Studied After Adrenalectomy and Oophorectomy No* 11-*deoxy* 17-*ketosteroids Were Present*

| Response to endocrine surgery | Patient | 11-deoxy 17-ketosteroids: E | A | β |
|---|---|---|---|---|
| Cancer regressed | As 17 | 0·03 | 0 | 0 |
| | As 9 | 0·01 | 0 | 0 |
| | As 7 | 0·04 | 0 | 0 |
| | As 38 | 0·01 | 0 | 0 |
| | As 26 | 0·01 | 0 | 0 |
| | Ax 4 | 0·02 | 0 | 0 |
| Cancer not improved | As 5 | 0·16 | 0·15 | 0 |

TABLE XI.—*The Excretion of Individual* 11-*deoxy* 17-*ketosteroids After Pituitary Implantation With* $^{90}Y$ *in* 13 *Women With Breast Cancer*

| Response to endocrine surgery | Patient | 11-deoxy 17-ketosteroids: E | A | β |
|---|---|---|---|---|
| Cancer regressed | Ys 14 | 0·20 | 0 | 0·10 |
| | S 3 | 0·83 | 0·19 | 1·20 |
| | Yu 10 | 0·26 | 0 | 0 |
| | Ys 6 | 0·45 | 0·35 | 0·14 |
| | Ys 3 | 0·07 | 0·02 | 0·27 |
| | Ys 1 | 0·09 | 0 | 0 |
| | Ys 22 | 0·04 | 0 | 0·01 |
| Cancer not improved | Ys 15 | 0·16 | 0·11 | 0 |
| | Ys 10 | 1·72 | 0·68 | 0 |
| | Ys 25 | 0·28 | 0 | 0·01 |
| | S 37 | 0·02 | 0·04 | 0 |
| | S 66 | 0·12 | 0·19 | 0·17 |
| | Ys 23 | 0 | 0 | 0·01 |

TABLE XII.—*Comparison of Mean Excretion of* 11-*deoxy* 17-*ketosteroids in* 12 *Patients Who Responded and* 10 *Patients Who did Not Respond to Pituitary Implantation*

| Response to endocrine surgery | Number of patients | Mean 11-deoxy-17-ketosteroids Arith. | Log. |
|---|---|---|---|
| Cancer regressed | 12 | 0·27 | 0·292 |
| Cancer not improved | 10 | 0·35 | 0·295 |
| Mean difference | — | 0·084 ± 0·250 | 0·003 ± 0·184 |
| *P* | — | >0·8 | >0·9 |

reported by Strong *et al.* (1956). In the adrenalectomized patient the small amounts of total 17-ketosteroids excreted in the urine are metabolites of maintenance cortisone, and if this is temporarily stopped they fall to undetectable levels (Hobkirk *et al.*, 1959).

In women with breast cancer whose adrenals have not been removed ACTH administration leads to increased excretion of urinary total 17-ketosteroids. This effect was not noted in any of the adrenalectomized patients studied, indicating that these patients were without functionally active adrenocortical tissue even when their cancer failed to respond, a finding which has recently been confirmed by more sensitive hormone assays (Sim *et al.*, unpublished).

Unlike adrenalectomy and oophorectomy, implantation of the pituitary with Yttrium-90 did not reduce total 17-ketosteroid excretion either in responding or non-responding patients. Atrophy of the adrenals after pituitary implantation is a gradual process and persistent adrenal steroidgenesis may account for the higher ketosteroid excretion compared with adrenalectomized patients (Forrest, Sim and Stewart, 1960).

Fractionation of the urinary neutral 17-ketosteroids provides a more sensitive and reliable estimate of adrenocortical function. Our results indicate that the pre-operative levels of these fractions are not of value in forecasting the response to endocrine surgery, a finding in agreement with that of Plantin *et al.*, (1958). Moreover, they do not confirm the recent suggestion by Allen, Hayward and Merivale (1957, 1958), that the ratio of 11-deoxy to 11-oxy 17-ketosteroids in the urine of patients with breast cancer is useful in this respect, although it must be stressed that these workers used methods of estimation different from ours and those of the Stockholm group.

After adrenalectomy and oophorectomy 11-oxy 17-ketosteroids are still present in the urine provided maintenance cortisone is taken, and they are of value in indicating persistent adrenocortical function only if maintenance cortisone is withdrawn (Hobkirk *et al.*, 1959 ; Sim *et al.*, unpublished). After pituitary implantation also, cortisone is required to maintain good health and metabolites will be excreted as 11-oxygenated steroids. It is therefore not surprising that the levels of 11-oxy 17-ketosteroids found in the urine after adrenalectomy and oophorectomy and after destruction of the pituitary bear no relationship to the clinical response.

The 11-deoxy 17-ketosteroids are mainly metabolites of the C19 androgenic steroids and their excretion is unlikely to be influenced by cortisone therapy (Holliday, Kellie and Wade, 1960). Their presence in the urine of women with breast cancer is thus highly suggestive of the presence of functioning adrenocortical, or in the case of aetiocholanolone, possibly ovarian tissue (Plantin *et al.*, 1958).

These steroids were completely absent from the urine of nine of sixteen patients after adrenalectomy and oophorectomy. In six of the remaining seven patients small amounts of aetiocholanolone (0·01–0·04 mg./24 hr.) only were detected and in only one patient whose cancer did not improve following adrenalectomy, were both aetiocholanolone (0·16 mg./24 hr.) and androsterone (0·15 mg./24 hr.) found. This latter patient did not have any clinical response to subsequent pituitary implantation with Yttrium-90 (100 per cent destruction) and no adrenal rests were found post mortem.

Other workers have also described small amounts of aetiocholanolone in the urine of adrenalectomized-oophorectomized patients (Kellie, 1954 ; Kellie and Wade, 1957 ; Bulbrook, Greenwood and Thomas, 1958). With the method we used the quantitative significance of amounts of less than 100 $\mu$g. is doubtful although they can be quantitatively detected down to a level approximating 10 $\mu$g. ; it is quite clear that the presence of such small amounts of this steroid in the urine of adrenalectomized patients does not preclude benefit from the operation.

Implantation of the pituitary with Yttrium-90 was less effective in reducing the excretion of these fractions and aetiocholanolone (0·02–1·72 mg./24 hr.) was found in the urine of twelve and androsterone (0·02–0·68 mg./24 hr.) and $\beta$ fraction (0·01–1·20 mg./24 hr) in the urine of seven of the twenty-two patients studied. The amounts of these steroids in the urine were not related to the clinical response.

Aetiocholanolone and androsterone have also been recovered from the urine of women with breast cancer after surgical hypophysectomy (Holliday, Kellie and Wade, 1958) although these workers could not isolate dehydroepiandrosterone from the urine of any of the twelve patients studied.

In our experience 11-deoxy 17-ketosteroids may still be recovered from the urine when the intrasellar pituitary is completely destroyed, although the levels are generally low provided 90 to 100 per cent of the gland is necrosed (Forrest *et al.*, 1960). It is known that aldosterone secretion persists despite complete hypophysectomy (McLean *et al.*, 1957) and evidence has also been presented that cortisol synthesis also may continue in the absence of a functioning pituitary gland (Forrest *et al.*, 1960). It does not seem unreasonable to suppose that the autonomous adrenal may also be able to synthesize C19 steroids, albeit in reduced amount, and further evidence of this has recently been reported by Wilson, Lipsett and Butler, (1960).

The presence of 11-deoxy 17-ketosteroids in the urine of women whose breast cancer had regressed following pituitary implantation clearly indicates that pituitary destruction can produce a beneficial response in the absence of full adrenal suppression. This would suggest that deprivation of a pituitary hormone other than corticotrophin was primarily responsible for its effect on tumour growth.

### SUMMARY

Urinary 17-ketosteroids and their fractions have been estimated in women with breast cancer before and after adrenalectomy and oophorectomy and implantation of the pituitary gland with Yttrium-90. No relationship has been found between pre-operative levels and the response to endocrine surgery. Following adrenalecomy and oophorectomy 17-ketosteroids with an 11-oxygen function, which are derived from maintenance cortisone, persist in the urine whereas those without this function are generally absent. Trace amounts of aetiocholanolone may still be found in the urine of some patients after adrenalectomy and oophorectomy but this is not of significance in relation to their response to the operation.

Following pituitary implantation with Yttrium-90 both 11-oxy and 11-deoxy 17-ketosteroids are more consistently found in the urine indicating that full adrenal suppression is not required for benefit from this operation.

We wish to thank Professor C. F. W. Illingworth in whose department this study was carried out, for his interest and guidance. This work was supported by the British Empire Cancer Campaign from whom Dr. Hobkirk, Mr. Sim, Mr. Blair and Dr. Stewart were in receipt of full-time grants. We also wish to thank Mr. R. A. McAllister, Miss Eleanor Lunney and Mr. Neal Carlin for technical assistance.

### REFERENCES

ALLEN, B. J., HAYWARD, J. L. AND MERIVALE, W. H. H.—(1957) *Lancet*, i, 496. (1958) 'Endocrine Aspects of Breast Cancer'. Edited by A. R. Currie. Edinburgh (Livingstone), p. 253.

BULBROOK, R. D., GREENWOOD, F. C. AND THOMAS, B. S.—(1958) *Biochem. J.*, **69**, 196.

FORREST, A. P. M., BLAIR, D. W., PEEBLES, BROWN, D. A., STEWART, HELEN J., SANDISON, A. T., HARRINGTON, R. W., VALENTINE, J. M., AND CARTER, P. T.—(1959) *Brit. J. Surg.*, **47**, 61.

*Idem*, SIM, A. W. AND STEWART, HELEN J.—(1960) *Proc. R. Soc. Med.*, **53**, 83.

HOBKIRK, R.—(1958) *J. clin. Endocrin.*, **18**, 636.

*Idem*, SIM, A. W., MCALLISTER, R. A., O'DONNELL, V. J., MORRIS, SASHA, PEEBLES BROWN, D. A., BLAIR, D. W. AND FORREST, A. P. M.—(1959) *Scot. med. J.*, **4**, 539.

HOLLIDAY, M. E., KELLIE, A. E., WADE, A. P.—(1958) 'Endocrine Aspects of Breast Cancer'. Edited by A. R. Currie. Edinburgh (Livingstone), p. 224.—(1960) *Acta Un. int. Cancr.*, **16**, 185.

KELLIE, A. E.—(1954) *Rep. Brit. Emp. Cancer Campgn.*, **32**, 464.

*Idem* AND WADE, A. P.—(1957) *Biochem. J.*, **66**, 196.

MCLEAN, J. P., LIPSETT, M. B., LI, M. C., WEST, C. D. AND PEARSON, O.H.—(1957) *J. clin. Endocrin.*, **17**, 346.

MEDICAL RESEARCH COUNCIL.—(1951) *Lancet*, ii, 29.

PLANTIN, L-O., BIRKE, G., DICZFALUSY, E., FRANKSSON, C., HELLSTROM, J., HULTBERT, S. AND WESTMAN, A.—(1958) 'Endocrine Aspects of Breast Cancer'. Edited by A. R. Currie. Edinburgh (Livingstone), p. 244.

STRONG, J. A., BROWN, J. B., BRUCE, J., DOUGLAS, M., KLOPPER, A. AND LORAINE, J. A.—(1956) *Lancet*, ii, 955.

WILSON, H.—(1954) *Arch. Biochem. Biophys.*, **52**, 217.

*Idem*, LIPSETT, M. B. AND BUTLER, L. C.—(1960) *J. clin. Endocrin.*, **20**, 534.